# Leader Humility, Sense of Power, and Interpersonal Deviance Relationship Model in the Bureaucratic Culture

**DOI:** 10.3389/fsoc.2022.929543

**Published:** 2022-07-08

**Authors:** Ambo Sakka Hadmar, Hendryadi Hendryadi, Suratna Suratna, Subur Karyatun

**Affiliations:** ^1^Management Department, Universitas Gunadarma, Depok, Indonesia; ^2^Sekolah Tinggi Ilmu Ekonomi Indonesia Jakarta, Jakarta, Indonesia; ^3^National Research and Innovation Agency Republic of Indonesia (BRIN), Jakarta, Indonesia; ^4^Economics and Business Faculty, Universitas Nasional, Jakarta, Indonesia

**Keywords:** leader humility, sense of power, interpersonal deviance, bureaucratic culture, Indonesia

## Abstract

**Purpose:**

Drawing on the approach—inhibition theory of power and the containment theory of control—we propose a relationship model of leader humility, sense of power, and interpersonal deviance, by placing bureaucratic culture as a boundary condition.

**Design/Methodology/Approach:**

Using a moderated mediation model, this study applies hierarchical regression and bootstrapping analyses to data obtained from online questionnaire responses of 428 employees from various sectors in Indonesia.

**Findings:**

The results reveal a positive relationship between leader humility and employees perceptions of the sense of power, as well as between the sense of power to interpersonal deviance. In addition, we confirmed the mediating role of the sense of power on the relationship between leader humility and interpersonal deviant. Bureaucratic culture has been confirmed to moderate the relationship between a sense of power and interpersonal deviance.

**Practical Implications:**

Companies can provide leadership training to leaders to convey to them when and where to demonstrate humility. Furthermore, the effectiveness of leaders' humility can be increased and their sense of power and interpersonal deviance reduced if the company adopts a low-level bureaucratic culture.

**Originality/Value:**

The current study contributes to the extant literature by revealing the moderating effects of bureaucratic culture on the relationship between the sense of power and interpersonal deviance, clarifying how, and when employees' sense of power stimulates interpersonal deviance in the Asian context.

## Introduction

In recent years, researchers have emphasized the need to identify and understand effective leadership. One concept that has attracted the attention of researchers is the theory of humble leadership, also known as leader humility (Owens, 2012). Leader humility is a part of the servant leadership philosophy and considered a characteristic of effective leadership (Greenleaf, 2002; Owens, 2012). Although leaders' humility has emerged as a significant issue in the areas of leadership and business ethics (Argandona, 2015; Frostenson, 2016; Lin et al., 2019), the use of leader humility is considered an exaggeration (Pfeffer, 2015). For example, Pfeffer (2015) argued that “humility is overrated and that we rarely observe humble leaders rise to the top” (see Qiuyun et al., 2020, p. 464). Furthermore, a humble leader can also act as a double-edged sword (Qin et al., 2020) for subordinates, have both positive and negative effects on employee behaviors (Oc et al., 2014; Qian et al., 2020; Qin et al., 2020; Qiuyun et al., 2020; Wang and Dust, 2021). Thus, understanding whether and precisely how leader humility affects employee behavior, as reported in earlier studies, requires further investigation (Qiuyun et al., 2020).

Leader humility is considered a core organizational virtue (Qiuyun et al., 2020) and can be interpreted differently in terms of concept and its effects on organizations, depending on their culture. Western scholars have empirically verified that leader humility is associated with job engagement (Owens et al., 2016) and individual and team performance (Owens, 2012; Rego, 2018). Similarly, non-Western scholars have also shown that leader humility contributes to improving psychological empowerment (Jeung, 2016), leader–member exchange (Qiuyun et al., 2020; Wang and Dust, 2021), and creativity (Hu et al., 2018). However, other evidence finds that humble leaders are believed to trigger subordinates' workplace deviance (Bharanitharan et al., 2019; Lin et al., 2019; Qin et al., 2020; Qiuyun et al., 2020). A noticeable omission in the existing body of research is the relationship between leader humility and subordinates' deviant behavior and how and when this relationship occurs (Qin et al., 2020). Thus, present study aims to expand the theoretical understanding of why humble leaders can have a negative effect on employees and when this tendency might be mitigated.

Accordingly, this study makes theoretical contributions in several ways. First, previous works have been tested to examine the relationship between leader humility and deviant employee behavior using multiple process models. For example, the links between leader humility and deviant behavior is mediated by psychological empowerment (Jeung, 2016), psychological entitlement, leader-member exchange (Jeung, 2016; Qin et al., 2020), and sense of power (Lin et al., 2019; Qiuyun et al., 2020). In contrast to previous studies which used a deviance behavior concept in general (Qiuyun et al., 2020), the present study focuses on interpersonal deviance includes various acts of incivility and violations of politeness norms (Pearson and Andersson, 2001).

Second, drawing on social information processing (SIP) theory (Salancik, 1978) and the approach-inhibition theory of power (Keltner and Gruenfeld, 2003; Anderson and John, 2012), we examine the mediating relationship between leader humility and interpersonal deviance through sense of power. As leaders' humble behavior fuels their subordinates' sense of power, subordinates with a high sense of power tend to engage in unethical behavior (Dubois and Rucker, 2015; Kennedy, 2017; Cho and Keltner, 2020). Indeed, it has been confirmed that the relationship between leader behavior and deviant employee behavior is mediated by sense of power (Lin et al., 2019; Qiuyun et al., 2020) in Chinese enterprises; the present study investigates a different background, namely Indonesia Enterprise. Since leader humility may be interpreted differently depending on culture, it is essential to examine its effect on interpersonal deviance to examine whether leader humility in Asian countries does not always have positive outcomes for organizations.

**Figure 1:**
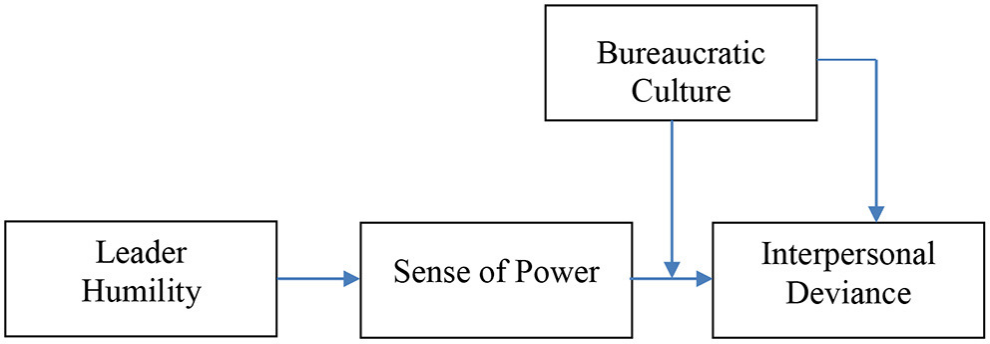
Hypothesized model.

Finally, as culture plays an essential role in the perception of leader humility (Oc et al., 2014) and leadership style in general (Hofstede and Hofstede, 2005), we propose that the effect of a sense of power on deviant behavior is moderated by organizational culture. We use the absence of social control or restraint assumptions to explain employees' deviant behavior (Clinard, 2015). We built and tested a model of the relationship between sense of power and employee interpersonal deviance to explore the underlying mechanisms and boundary conditions (see Figure 1), thereby advance the bureaucratic culture literature and enriching our understanding of the conditional effect of sense of power on interpersonal deviance by empirically exploring the moderating effect of bureaucratic culture.

## Theoretical Framework and Hypotheses

We use the social information processing (SIP) theory (Salancik, 1978) and the approach-inhibition theory of power (Keltner and Gruenfeld, 2003; Anderson and John, 2012) as theoretical frameworks to explain the impact of a humble leader on the behavior of subordinates. The specific reasons for using these two theories are as follows. First, SIP describes individuals as organisms that can adapt their behavior to the environment based on the information they receive. Based on the premise of the SIP, humble behavior toward subordinates, such as recognition of their strengths and contributions as well as an openness to new ideas and feedback, is a signal to subordinates that their contributions are valued, and that the leader needs them. Indeed, subordinates translate the humble behavior of a leader as a role model that can trigger subordinates' humble behavior (Rego et al., 2017; Nielsen, 2018; Diao et al., 2019; Zhong et al., 2020). On the other hand, a humble leader can also increase perceived psychological empowerment (Jeung, 2016), employee confidence in their abilities (Chiu et al., 2016), and sense of power (Lin et al., 2019; Qin et al., 2020; Qiuyun et al., 2020). Moreover, SIP has been used by previous researchers to explain the impact of the environment on deviant behavior (Martel, 2019), particularly the relationship between leadership behavior and subordinate deviant behavior (Aryati et al., 2018; Qin et al., 2020).

Second, leader humility can also increase employees' sense of power (Lin et al., 2019; Qin et al., 2020; Qiuyun et al., 2020), and at the same time, a sense of power can increase subordinates' potential for deviant behavior (Qiuyun et al., 2020). An alternative explanation of the relationship model relies upon the approach-inhibition theory of power (Keltner and Gruenfeld, 2003). This study also examines bureaucratic culture as a situational factor in the relationship between sense of power and deviant behavior. From a behavioral perspective, Owens (2012) conceptualized leader humility as an interpersonal characteristic consisting of a desire to see oneself accurately, recognizing the strengths and contributions of others, and opening up to ideas and feedback. Since a humble leader is more focused toward building positive relationships with subordinates, the expected consequence is a higher-quality leader-follower relationship, which results in a greater commitment from subordinates to contribute to the group (Owens and Johnson, 2013). A humble leader willing to admit his/her mistakes and weaknesses can trigger subordinates' doubts about his/her abilities. Instead of receiving rewards from subordinates, this type of leader triggers workplace and organizational deviance (Qin et al., 2020; Qiuyun et al., 2020) and contradictory voice behavior (Lin et al., 2017; Bharanitharan et al., 2019).

### Leader Humility and Sense of Power

Sense of power is an individuals' ability to influence another person or other people in interpersonal relationships within their environment, including co-workers, friends, family, and romantic partners (Keltner and Gruenfeld, 2003). In short, a sense of power is related to a social relation that reflects one's influence on other individuals' attitudes and behaviors and can be understood from the relationship between one person to another (Morrison and See, 2015). In the same vein, according to SIP theory (Salancik, 1978), leadership is an essential source of information for subordinates and a role model. A humble leader sends a salient message to followers that they are valued and empowered. Of course, the behavior of leaders willing to listen to employee input and ideas will be interpreted by employees as a form of pride that encourages more significant contributions in the future. Furthermore, when the leader can create a situation in which the strengths and contributions of subordinates are recognized, employees adjust the situation by tending to dare to convey input and creative ideas. The nature of a humble leader who recognizes the contribution and empowers subordinates' abilities is a positive signal that triggers confidence in their abilities (Chiu et al., 2016) and a sense of power (Lin et al., 2019; Qin et al., 2020; Qiuyun et al., 2020). Moreover, the relationship between leader humility and sense of power has been documented in previous studies (Lin et al., 2019; Qiuyun et al., 2020). Drawing on this theoretical paradigm, we suggest that employees who feel empowered by their leaders, will feel a greater sense of power. Thus, we posit the following hypotheses:

H1. Leader humility is positively correlated with employee sense of power.

### Sense of Power and Interpersonal Deviance

According to Pearson and Andersson (2001), interpersonal deviance such as verbal and non-verbal abuse, offensive jokes or comments, and forms of speech that lead to ethnic or racial slurs, are aimed at organization members (Ferguson, 2011). In general, the form of interpersonal deviance includes various acts of incivility and violations of politeness norms (Pearson and Andersson, 2001). The link between sense of power and interpersonal deviance is possible because the approach-inhibition theory of power asserts that employees who have a sense of power engage in deviant behavior. The theory argues that employees with a high sense of informal power obtained from leadership recognition, may lead individuals to act based on personal desires without consideration of social norms (Keltner and Gruenfeld, 2003; Galinsky et al., 2008; Pitesa, 2012). For example, people who have a high sense of power tend to engage in abusive supervision (Hussain, 2017; Park et al., 2019), sexual harassment (Kunstman, 2011). Recently, Qiuyun et al. (2020) also found a positive effect of a sense of power on deviant behavior. Thus, we posit the following hypotheses:

H2. Employee sense of power is positively correlated with interpersonal deviance.

### The Mediating Effect of Sense of Power

The relationship between leader humility and deviant behavior was demonstrated as a process model based on previous studies. For example, the relationship between leader behavior and deviant employee behavior is mediated by confidence (Qiuyun et al., 2020), psychological empowerment (Jeung, 2016), psychological entitlement, leader-member exchange (Qin et al., 2020), and sense of power (Lin et al., 2019; Qiuyun et al., 2020). Qin et al. (2020) use the term “the double-edged sword of leader humility” to express the multi-role leader humility in the Asian cultural. In drawing from the attribution theory, they found that leader humility positively affects subordinates' psychological entitlement, which in turn increases workplace deviance. Conversely, when subordinates do not attribute leader humility in a self-serving manner, leader humility is positively associated with the leader-member exchange, thus decreasing workplace deviance (Qin et al., 2020). As such, we propose that personal sense of power as a subordinates' perception of their ability to influence others (Lin et al., 2019) can represent the subjective feeling of power that comes from the relationship between leaders and followers. On this basis, we argue that leader humility is effective to facilitate interpersonal deviant by instilling in employees a greater sense of power. Previously, a high sense of power was not always associated with positive attitudes (e.g., employee voice, Lin et al., 2019), but also negative things, including abusive behavior (Hussain, 2017; Park et al., 2019) and sexual harassment (Kunstman, 2011). Drawing from the approach-inhibition theory of power and empirical literature, we argue that leader humility is related to employee interpersonal deviance by enhancing the employees' sense of power.

H3. Leader humility is positively correlated with interpersonal deviance through a sense of power.

### The Role of Bureaucratic Culture

The term “bureaucracy” initially referred to governing officials (bureaucrats) holding political power and exercising governmental decision-making; Weber (1979) expands the form of bureaucracy into various types, including public administration, government, and business. Weber (1979) provides a list of the characteristics of bureaucracy, including hierarchical structure, task division, formal rules, and regulation, that generally describe a general feature of public administration (Claver et al., 1999). The present study focuses on a specific culture, namely, a bureaucratic culture with the following characteristics: centralized decision-making, high degree of control, top-down communication, individual searches for stability, and change resistance (Hofstede and Hofstede, 2005; Hendryadi et al., 2019). Although this culture tends to be present in governmental organizations, it can also be adopted by business organizations, especially in developing countries (Hofstede and Hofstede, 2005) and Indonesia is no exception. Indonesian national culture is described as a high-power distance, which shows hierarchy, unequal rights between power and non-power holders, and leader inaccessibility (Hofstede and Hofstede, 2005). Furthermore, leaders are seen as directives, high management controls, and delegates which is congruent with bureaucratic culture (Wallach, 1983).

In general, culture can be formed from a combination of geographical, historical, and local or cultural countries; organizations operating in the West and East have cultural differences. Painter (2010) specifically discuss the differences in bureaucratic culture in nine different regions (e.g., Anglo-American, East Asia, Napoleonic, Islamic). For example, East Asian countries such as Japan interpret bureaucratic culture based on local traditions. In contrast, Thai bureaucratic culture is formed by Buddhist norms and patrimonial social structures (Painter, 2010). Other countries, such as Malaysia and Indonesia, have a different history of colonization, making them slightly different in translating bureaucratic culture, even though they are both influenced by norms originating from Islam (Painter, 2010).

This study uses an approach-inhibition theory of power (Keltner and Gruenfeld, 2003) and containment theory (Reckless, 1973) as a theoretical basis for explaining the role of bureaucratic culture in the relationship between leader humility, sense of power, and deviant behavior. Since many researchers have tested control theories on other types of deviant behavior, where outer containment was related to avoiding deviant behavior (Kennedy, 2017), we propose bureaucratic culture as the outer containment to explain the variation of interpersonal deviance. A hierarchy system, control, and power distance are inherent features of a bureaucratic culture will have positive consequences on the relationship of sense of power and interpersonal deviant. For example, Soral and Arayankalam (2020) found a positive link between bureaucratic culture and deviant behavior. Similarly, Hussain (2017) also found that high power distance orientation can amplify the effect of abusive supervision on workplace deviance. Going beyond this main effect found in past research (Hussain, 2017; Soral and Arayankalam, 2020), we argue that bureaucratic culture with high-power distance, centralized control, and formal hierarchy is a boundary condition in the sense of power-employee deviant behavior relationships.

H4. Bureaucratic culture is positively correlated with interpersonal deviance.
H5. Bureaucratic culture moderates the relationship between an employee's sense of power and interpersonal deviance.

## Methodology

### Sample and Procedure

In June 2020, this study adopted purposive sampling to select participants from various companies through the alumni networks of three private universities in Jakarta. We contacted alumni who were enrolled in the alumni center and invited them to participate in research activities. The study included representatives of companies who were willing to be involved in the survey, and they in turn asked other groups and employee communities if they wanted to be involved in this study. The respondents were informed about the objective of the survey, the voluntary nature of participation, and the procedures for completing the online questionnaires. They were assured of the confidentiality of their responses. Identification codes were used to match the participants' survey responses across the two phases to ensure confidentiality.

The data for this study were collected through the means of the online survey method (the survey was split into two phases: phase I and phase II). In phase I, in addition to reporting demographic information, the participants answered questions regarding leader humility and perceived personal sense of power. A total of 521 employees participated in this phase. After excluding 59 unqualified responses, 462 complete and usable questionnaire responses were obtained. Approximately 2 months later, a second questionnaire was sent to the participants of phase I. In phase II, the respondents were asked to answer questions regarding their personal sense of power and interpersonal deviant behavior. Similar to phase I, data were collected during the COVID-19 pandemic period from August to September 2020. After eliminating unqualified responses, the final sample considered for this study consisted of 428 questionnaire responses, with a final overall response rate of 93% from phase 1.

The respondents were identified as working in various sectors, such as the financial services sector (39.05%), public sector/government (32.45%), and manufacturing sector (19.79%). The remaining 8.71% of the respondents did not state the details regarding their organization. Among the respondents, there were 265 men (62%) and 163 women (38%). Regarding their educational backgrounds, 48.28% of the respondents had completed a bachelor's degree, 28.76% had a diploma, 15.30% had a high school diploma, and 7.65% had a master's degree. Furthermore, 3.7% of the respondents were under the age of 30, 10.8% between 31 and 35, 45% between 36 and 45, and 40% over the age of 45. The average age of the respondents was 29 years, and the average work experience of the respondents was between 2 and 5 years (42.70%).

### Measurement

All items were rated on a five-point Likert scale, ranging from 1 (strongly disagree) to 5 (strongly agree). A 9-item scale was used to measure leader humility (Owens, 2012), with employees evaluating their leader's humility. This scale has been widely used in the Asian countries (e.g., Qin et al., 2020; Qiuyun et al., 2020). Sample items included “My supervisor actively seeks feedback, even if it is critical,” “My supervisor admits it when he or she does not know how to do something,” “My supervisor acknowledges when others have more knowledge or skills than him or her,” and “My supervisor is open to others' ideas.” The scale's reliability was 0.88.

The sense of power was measured via a self-reported 8-item scale developed by Anderson and John, 2012. Some sample items included, “I think I have a great deal of power” and “If I want to, I get to make the decision.” The scale's reliability was 0.85.

The measure for bureaucratic culture was adapted from a 5-item scale developed by Hendryadi et al. (2019). The respondents were asked to give ratings from 1 (*low*) to 5 (*high*) on items such as centralized decision-making, high degree of control, top-down communication, individual search for stability, and change-resistant. A high score indicates a high level of bureaucratic culture in the company. The scale's reliability was 0.92.

Interpersonal deviance was measured by using a 7-item scale developed by Bennett (2000). The respondents were asked to answer questions regarding their interpersonal deviant behavior in the past 6 months. The sample items included the following: “Publicly embarrassed someone at work,” “Cursed at someone at work,” and “Made fun of someone at work.” The scale's reliability was 0.87.

Control variable*-* since perceived leader behavior, sense of power, and deviant behavior are closely related to demographics (Santos, 2014; Wang and Dust, 2021), we controlled these demographic variables. In our data analysis: gender (female = 0 and male = 1), age (<20 years = 1, 21–30 years = 2, 31–40 years = 3, above 40 years = 4), and tenure (<2 years =1, 2–5 years = 2, >5 years = 3).

### Hypothesis Testing Strategy

The hypotheses were tested using a moderation-mediation model (Hayes, 2017). Regarding the five hypotheses proposed in this study, a multi-step regression analysis was executed using PROCESS (version 4.0), a macro developed (model 14) by Hayes (2017). These models were tested using the bootstrapping technique (Hayes, 2017), which provides a more reliable estimation of indirect effects and does not make assumptions regarding the normality of the sampling distribution, which is often unrealistic. Significant results were identified by examining 95% bias-corrected confidence intervals calculated based on 5,000 bootstrapped samples. A confidence interval that does not include zero indicates a significant mediation or moderation effect.

## Results and Discussion

### Common Method Bias and Confirmatory Factor Analysis

This study collects data from a single source which has the opportunity to be exposed to the problem of common methods bias (Podsakoff et al., 2003). To ensure that the data is free from common method bias, we carry out a series of procedures to remedy it. First, we ensure that the questionnaire is anonymous to increase the objectivity of respondents' answers. Furthermore, the common method variance (CMV) was examined by using full collinearity test using PLS-SEM (Kock, 2017). The results from the full collinearity test (see Table 1) show that there is no item having a variance inflation factor (VIF) > 3.3, which confirms that CMV is not a significant threat to this data (Kock, 2017).

**Table 1:** Scale items and evaluation of the measurement model.

**Variable**	**Factor loading**	**VIF**	**CA**	**CR**	**AVE**
Leader humility	0.52–0.78	1.27–2.42	0.88	0.90	0.51
Sense of power	0.63–0.79	1.32–2.21	0.85	0.88	0.52
Bureaucratic culture	0.79–0.90	2.18–3.26	0.92	0.93	0.74
Interpersonal Deviant	0.71–0.82	1.56–2.34	0.87	0.90	0.56

*VIF, Variance Inflation Factor; CA, Cronbach Alpha; CR, Construct Reliability; AVE, Average Variance Explained*.

To assess the measurements' construct validity, convergent, and discriminant validity were tested. Convergent validity was indicated by using factor loadings, composite reliability (CR), and average variance extracted (AVE). The evaluation of the measurement model was based on the factor loading value with a cut-off value of standardized factor loadings >0.50 (Hair et al., 2010). The results show that all the indicators have adequate validity (factor loading value > 0.50). The composite reliability illustrates the extent to which construct indicators ranging from 0.88 to 0.93. The results exceeded the recommended value of 0.7 (Hair et al., 2010). The average variance extracted (AVE) reflects the total number of variants in the indicator representing latent constructs. Table 1 shows the AVE values ranging from 0.51 to 0.74, also exceeding the recommended threshold of 0.50, providing support for convergent validity (Hair et al., 2010). Discriminant validity was evaluated by comparing the root square value of AVE with all correlations between variables (Fornell and Larcker, 1981). Table 2 shows that all values of the root square of AVEs (diagonal bold italic) are greater than the correlations between variables, which indicates that discriminant validity has been met.

**Table 2:** Descriptive statistics and correlations of the study variables.

**No**		**Mean**	**S.D**	**1**	**2**	**3**	**4**	**5**	**6**	**7**
1	Gender	1.51	0.50	1						
2	Education	1.80	0.75	−0.01	1					
3	Tenure	1.50	0.50	−0.05	−0.01	1				
4	LDH	3.92	0.65	−0.04	−0.06	−0.07	***0.72***			
5	SOP	3.68	0.66	−0.02	0.02	−0.03	0.37^**^	***0.72***		
6	BRC	3.32	0.92	0.00	0.02	−0.03	−0.08	−0.16^**^	***0.86***	
7	DEV	2.50	0.71	0.03	0.02	−0.04	0.16^**^	0.15^**^	0.32^**^	***0.75***

*S.D, Standard deviation, average variance extracted (AVE) = diagonal bold italic. The correlations are based on N = 428*;

** *p < 0.01*,

* *p < 0.05*.

### Descriptive Statistics

The findings (see Table 2) showed that leader humility was positively correlated with sense of power (*r* = 0.34, *p* < 0.01) and interpersonal deviance (*r* = 0.16, *p* < 0.01). Leader humility was found to have a negative and insignificant relation to bureaucratic culture (*r* = −0.08, *p* > 0.05), while sense of power was positively correlated with bureaucratic culture (*r* = 0.16, *p* < 0.01). Bureaucratic culture had a significant positive correlation with interpersonal deviance (*r* = 0.32, *p* < 0.01).

### Hypothesis Testing

H1 stated that leader humility is positively related to sense of power, and this hypothesis was supported because the regression coefficient showed that leader humility and sense of power were positively associated (β = 0.52, *p* < 0.01) (see Table 3), which is also consistent with the results of the correlation analysis. H2 stated that the sense of power is positively related to individual deviance, and the results supporting H2 are statistically significant (β = 0.28, *p* < 0.01). Consistent with H2, the results show that the role of the sense of power mediates the relationship between leader humility and interpersonal deviance (see Table 4). Additionally, the bootstrapping estimation of the indirect effect of leader humility on interpersonal deviance was positive and significant (the confidence interval [CI] using a 5000-bootstrap sample does not include 0; LLCI = 0.03, ULCI = 0.13). H4 stated that the bureaucratic culture is positively related to interpersonal deviance, and the results are statistically significant (β = 0.19, *p* < 0.01). Thus, H1–H4 was supported.

**Table 3:** Results of the mediated-moderated analyses (PROCESS: model 14).

**Outcome: sense of power**	**β**	***S.E***	***P***	***LLCI***	***ULCI***
Gender	−0.01	0.06	0.93	−0.12	0.11
Education	0.03	0.04	0.38	−0.04	0.11
Tenure	−0.01	0.04	0.85	−0.09	0.07
LDH	0.38	0.05	0.00	0.29	0.47
Outcome: interpersonal deviance	β	*S.E*	*P*	*LLCI*	*ULCI*
Gender	0.06	0.06	0.38	−0.07	0.18
Education	0.01	0.04	0.88	−0.07	0.08
Tenure	−0.01	0.04	0.76	−0.10	0.07
LDH	0.14	0.05	0.01	0.03	0.24
BRC	0.19	0.05	0.00	0.09	0.29
SoP	0.29	0.04	0.00	0.23	0.36
Interaction	0.18	0.05	0.00	0.08	0.27

*N, 428. Bootstrap sample size = 5,000. S.E, Standard Error; LDH, Leader humility; BRC, Bureaucratic culture; SpP, Sense of Power; DEV, Interpersonal deviance; Interaction, SoP x BRC*.

**Table 4:** Conditional moderating and mediating effect.

**Conditional effects of the focal predictor at values of the moderator**	***Effect***	***SE***	***LLCI***	***ULCI***	***p***
Low BRC	0.03	0.07	−0.10	0.16	0.65
High BRC	0.35	0.07	0.21	0.49	0.00
Indirect effect	*Effect*	*S.E*	*LLCI*	*ULCI*	
LDH > SOP > DEV	0.07	0.03	0.03	0.13	
Index of moderated mediation	Index	*S.E*	*LLCI*	*ULCI*	
LDH > SOP > DEV	0.07	0.03	0.02	0.12	

*N = 428. Bootstrap sample size = 5,000. S.E, Standard Error; LL, lower limit; CI, confidence interval; UL, upper limit; LH, leader humility; BRC, bureaucratic culture; SOP, Sense of Power*.

H5 stated that bureaucratic culture moderates the relationship between the sense of power and interpersonal deviance. As shown in Table 4, the interaction term (sense of power × bureaucratic culture) shows a positive and significant parameter (β = 0.18, *p* < 0.05). This means that bureaucratic culture plays a moderating role in the relationship between the sense of power and interpersonal deviance.

**Figure 2:**
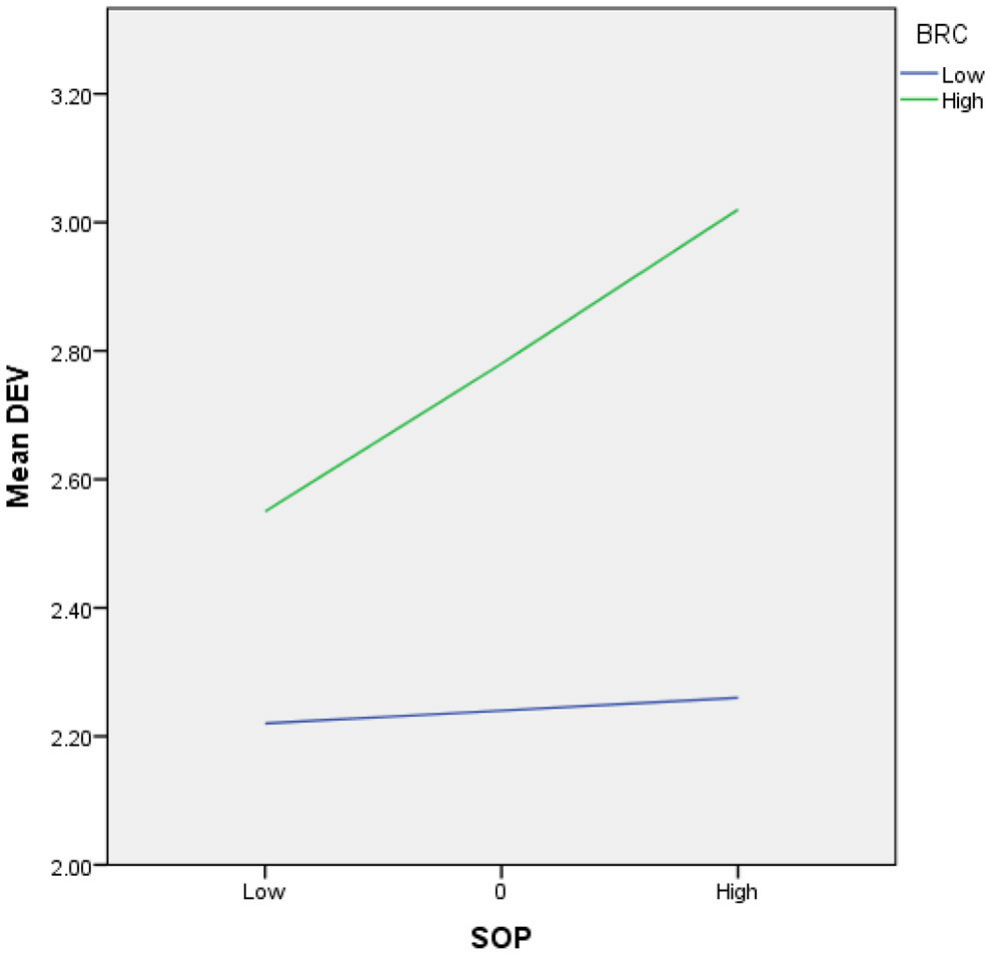
Moderating effect of bureaucratic culture on the relationship between the sense of power and interpersonal deviance.

Furthermore, we used Aiken et al. (1991) procedure of simple slopes at higher and lower levels of bureaucratic culture (one standard deviation above and below the mean) to plot the interaction. Table 4 and Figure 2 shows that the positive effect of the sense of power on interpersonal deviance was significant for high levels of bureaucratic culture (β = 0.35, *p* < 0.01). However, for low-level bureaucratic culture, the effect of the sense of power on interpersonal was insignificant (β = 0.03, *p* > 0.05). Additionally, the bootstrapping estimation of the direct effect of leader humility on employee sense of power was positive and significant only for high-level bureaucratic culture (the confidence interval [CI] using a 5000-bootstrap sample does not include 0; LLCI = 0.21, ULCI = 0.49).

## Discussion and Implication

The main aim of this study was to explore how and when leader humility fosters a sense of power among employees, examining the mediating role of employees' sense of power in the relationship between leader humility and interpersonal deviance, as well as the role of bureaucratic culture as moderator in these relationships. The results derived from the data collected from 428 employees from various companies in Indonesia indicate that leader humility is positively related to employees' sense of power and interpersonal deviance via the sense of power. Furthermore, an employee's sense of power and bureaucratic culture can trigger employees' interpersonal deviant.

### Theoretical Implications

Taken together, we believe that the present findings generally support our application of the approach-inhibition theory of power to the relationship between leader humility and sense of power, and sense of power and interpersonal deviance, with certain caveats. First, our finding that the positive effect of leader humility on the sense of power aligns with the elevated power that arises from the humble behavior of the leader at work could prompt individuals to perceive that their leader provides a lot of support and recognition for contributions, which could lead to enhanced positive responses from their leaders. Our study supports the relationship between leader humility and a sense of power, which is in line with previous studies (Lin et al., 2017; Qin et al., 2020; Qiuyun et al., 2020). Our study broadens these studies in different cultures (Owens, 2012).

Second, our findings support the approach-inhibition theory of power, which assumes that an increase in the sense of power has both positive and negative consequences (Cho and Keltner, 2020). This study thus proves the negative trend where the informal power of employees impacts their sense of power as a response to humble leader behavior, and subsequently, how this high sense of power leads to a tendency for deviant behavior. In addition, earlier studies have explored deviant behavior in various forms, such as inducing challenging voices (Bharanitharan et al., 2019), organizational deviance (Qin et al., 2020), and subordinate workplace deviance (Qiuyun et al., 2020). Thus, we extend previous studies by explicitly exploring interpersonal deviance. More broadly, these findings support the idea that leader humility in Asian countries does not always have good outcomes for organizations. In other words, an increase in the sense of power obtained from the humble behavior of the leader causes employees to behave negatively toward their co-workers.

Third, and more importantly, the bureaucratic culture in this study acts as a quasi-moderator. First, bureaucratic culture is positively related to interpersonal deviance, indicating that high levels of bureaucratic culture create opportunities for interpersonal deviance in the workplace. Our findings confirm the findings of Soral and Arayankalam (2020) regarding the positive relationship between bureaucratic culture and deviant behavior (e.g., cyberloafing). Second, we examined the role of bureaucratic culture as a boundary condition for the relationship between sense of power and interpersonal deviance. The first situation, the bureaucratic culture, has a moderating role in the sense of power and interpersonal deviance relationship, where the relationship is more robust in the high-level bureaucratic culture. This condition indicates that a high level of bureaucratic culture can increase the effect of sense of power on interpersonal deviance, and vice versa when the bureaucratic culture is at a low level, the relationship becomes insignificant.

Finally, our study also contributes to the SIP theory and approach-inhibition theory of power and containment theory to explain deviant behavior. We contribute to the SIP theory by integrating it with the approach-inhibition theory of power to broaden our understanding of the undesirable impact of leader humility on employee behavior (e.g., deviant behavior). The SIP theory states that subordinates will adapt to the information given them and their environment, which can effectively explain the relationship between leader humility and a sense of power. By applying the approach-inhibition theory of power, we find that a high sense of power caused by leader humility can have a continuing effect on the interpersonal deviance of subordinates. Moreover, our study also helps enrich scholarly understanding of the conditional effect of sense of power on interpersonal deviance by empirically exploring the moderating effect of bureaucratic culture.

### Practical Implications

The practical implications of this study are as follows: Principally, given the finding that leader humility can predict a sense of power, and in turn, that a high sense of power can have negative consequences on interpersonal deviance, managers should be mindful of how to exhibit effective humble leadership. Although this study found that leader humility has positive effects on employees (e.g., sense of power), subsequent effects on interpersonal deviants need to be anticipated. Thus, companies can provide leadership training that helps leaders demonstrate humility by considering their situation and time. If the goal is to increase the sense of power, which may be helpful for motivated employees, to increase their focus on task demands, job satisfaction, performance, OCB, and job engagement (Owens et al., 2016; Qiuyun et al., 2020), companies must ensure that the adopted culture is less bureaucratic. In addition, the application of low-level bureaucratic culture seems to be key to maintaining humble leadership on track, namely, to increase the sense of power and eliminate its adverse effects on interpersonal deviance.

Human resource practices should adopt a different approach to prevent and reduce deviant behavior for organizations that adopt a bureaucratic culture (e.g., government organizations), since the adverse effects of a sense of power on the interpersonal deviance of employees increase in organizations that have a high bureaucratic culture. Hence, employee training or development programs required to reduce interpersonal deviance will differ between bureaucratic and non-bureaucratic organizations. For example, in a business organization with a flat structure, employees should ideally receive training to express ideas openly and reciprocate effective communication with their superiors. In contrast, employees should ideally receive training to use formal and structured communication patterns based on predefined hierarchical lines for government organizations that rely on a high-level bureaucratic structure and power distribution.

### Limitations and Future Research Directions

This study has several limitations that future researchers should bear in mind. First, the data used to test the hypotheses were obtained via purposive sampling using a cross-sectional design, which may limit generalizability and the determination of causality, as well as posing the risk of common method bias (Podsakoff et al., 2012). Although we used a time-lag design in data collection to control for common method bias, interpretations of the causal relationships between variables remain limited. A random sampling design should also be applied to improve the generalizability of the results.

Second, we realize that the CMV is unlikely to be excluded entirely for single-source data; the factor analysis results in this study reduced the concern regarding CMV. In addition, this study used a time-lag design to minimize the CMV risk (Podsakoff et al., 2012; Law et al., 2016); and we believe that the results are less likely to be biased. Future studies should adopt longitudinal data collection and random sampling approaches to strengthen the claims of the causal model proposed in this study. Moreover, it is essential for future researchers to consider the potential cluster effect of organizational units or organizational control variables (such as unit size) on the reliability of the analysis. We suggest that future studies apply a multigroup analysis design to ascertain differences in results between groups.

Third, this study was conducted during the COVID-19 pandemic, thereby reducing social interaction between employees due to work from home policy. Although the respondents were asked to answer questions regarding their interpersonal deviance in the past 6 months, conducting a comparative study in normal situations is necessary.

Finally, we explored the mediating role of employee sense of power and the moderating role of bureaucratic culture. Future studies might determine alternative factors that link leader humility to interpersonal deviance. For example, leader humility may improve employee humility (Zhong et al., 2020), leader-member exchange and subordinate psychological entitlement (Qin et al., 2020), and psychological empowerment (Jeung, 2016). Future research can also reexamine the moderating role of bureaucratic culture in these models.

## Data Availability Statement

The original contributions presented in the study are included in the article/supplementary material, further inquiries can be directed to the corresponding authors.

## Ethics Statement

Ethical review and approval was not required for the study on human participants in accordance with the local legislation and institutional requirements. Written informed consent for participation was not required for this study in accordance with the national legislation and the institutional requirements.

## Author Contributions

AH: conceptualization. HH: methodology. HH, SS, and SK: formal analysis. AH and SK: data curation. AH and HH: writing—original draft preparation. SK and SS: writing—review and editing. All authors read and approved the final manuscript.

## Conflict of Interest

The authors declare that the research was conducted in the absence of any commercial or financial relationships that could be construed as a potential conflict of interest.

## Publisher's Note

All claims expressed in this article are solely those of the authors and do not necessarily represent those of their affiliated organizations, or those of the publisher, the editors and the reviewers. Any product that may be evaluated in this article, or claim that may be made by its manufacturer, is not guaranteed or endorsed by the publisher.
